# Fungal peritonitis in children on peritoneal dialysis at a tertiary care Centre

**DOI:** 10.1186/s12882-020-02014-1

**Published:** 2020-09-16

**Authors:** Mohammed Alsuhaibani, Egab Aldosari, Khawla A. Rahim, Saeed Alzabli, Dayel Alshahrani

**Affiliations:** 1 Department of Paediatrics, College of Medicine, Qassim University, P.O. Box 6666, Buraidah, Qassim 51452 Saudi Arabia; 2 Department of Paediatric Infectious Diseases, King Fahad Medical City, Riyadh, Saudi Arabia; 3 General Paediatric Department, King Fahad Medical City, Riyadh, Saudi Arabia; 4 Department of Paediatric Nephrology, King Fahad Medical City, Riyadh, Saudi Arabia

**Keywords:** Peritoneal, Nephrotic, Candida, Catheter, Dialysis, Fungal

## Abstract

**Background:**

Fungal peritonitis (FP) is an infrequent but serious complication in children undergoing peritoneal dialysis (PD). This study aimed to explore the risk factors, clinical manifestations, causative organisms, fungal susceptibility findings, and outcomes of FP in children from Saudi Arabia.

**Methods:**

In this case–control study, the medical records and laboratory results of paediatric patients aged 0–14 years who underwent PD were reviewed for FP episodes. All FP episodes were matched with PD-related bacterial peritonitis episodes (1:4 ratio).

**Results:**

A total of 194 episodes of PD-related peritonitis occurred between 2007 and 2017, among which 11 were FP episodes (5.6%), representing a rate of 0.03 episodes per patient-year. Of these 11 episodes, 9 were caused by *Candida* species (82%). Compared with the bacterial peritonitis group, the FP group had a higher proportion of patients with congenital/infantile nephrotic syndrome (*p* = 0.005) and those younger than 5 years of age (*p* = 0.001). We observed a higher rate of catheter removal in the FP group than in the bacterial peritonitis group (*p* <  0.001); however, 1 patient died despite catheter removal. Moreover, 75% of *Candida* species isolates were susceptible to fluconazole.

**Conclusions:**

This study revealed that FP is associated with a significant risk of peritoneal membrane failure among children undergoing PD. Therefore, early diagnosis and prompt management are essential. We also found that congenital/infantile nephrotic syndrome and young age (5 years old or younger) were risk factors for FP in children undergoing PD.

## Background

Peritoneal dialysis (PD) is the best procedure for children with end-stage renal disease. Peritonitis is the most common complication in patients on PD, with a higher frequency in children than in adults, and it is the most frequent cause of treatment failure and mortality in patients on PD [1, 2].

The causative pathogens and patient susceptibility to PD-related peritonitis vary from time to time and from region to region; hence, the International Society for Peritoneal Dialysis (ISPD) endorses the use of empirical antimicrobial medication to treat PD-related peritonitis depending on the region [3]. In children, PD-related bacterial peritonitis is the most frequently identified form of peritonitis, while peritonitis caused by fungal organisms account for 2–12.9% of all cases. PD-related fungal peritonitis (FP) has a higher morbidity and mortality than bacterial peritonitis. *Candida* species, especially the non-*albicans* species, are the most frequent organisms causing PD-related FP in adults and children [4]. Peritonitis caused by fungi such as *Aspergillus*, *Fusarium*, *Rhodotorula*, *Mucorales*, and dematiaceous moulds were reported periodically [5, 6]. PD-related FP could be a consequence of unsterile techniques during PD preparation, catheter exit-site infection, intestinal perforation, or fistulae between the genitourinary/gastrointestinal system and the peritoneum. Exposure to bacterial infection and recent use of antibiotics have also been considered as risk factors, especially if the exposure is within 30 days of an FP episode. Age is another risk factor: children below the age of 2 years are at a greater risk of developing FP [4]. Moreover, prolonged hospitalization and poor outcomes, such as peritoneal membrane failure, are associated with FP in paediatric patients undergoing PD [7–9].

In Saudi Arabia, there have been few studies on PD-related peritonitis and its outcomes in children. PD-related FP has been identified in up to 8% of total peritonitis cases in local observational studies [10–12]. This study aimed to explore the risk factors, clinical manifestations, causative organisms, fungal susceptibility findings, and outcomes of FP in paediatric patients who underwent PD at a tertiary care centre in Saudi Arabia.

## Methods

This was a retrospective case–control study of all episodes of FP occurring in children aged 0–14 years who underwent PD between January 2007 and December 2017 at King Fahad Medical City, Riyadh, Saudi Arabia. Electronic medical records were reviewed for demographic data, age at peritonitis episode, sex, primary diagnosis, number of peritonitis episodes, prior antibiotic use, clinical manifestation, dialysate analysis, causative fungal species, susceptibility results, outcomes of peritonitis, complete blood count, and serum electrolyte levels. The diagnosis of PD-related peritonitis was defined based on the 2016 International Society For Peritoneal Dialysis ISPD recommendation [3], and confirmed when at least 2 of the following characteristics were present: (1) manifestation compatible with peritonitis (abdominal pain and/or cloudy dialysis effluent); (2) effluent dialysis leukocyte count greater than 100/μL (at least 2 h after a dwell time) with more than 50% of polymorphonuclear cells; and (3) a positive culture from dialysis effluent.

In the laboratory, peritoneal dialysis effluent samples were tested for white blood cell count, differential count, and Gram staining, and were then inoculated into BACTEC Peds Plus/F media (BD BACTEC FX Blood Culture Instrument; Becton Dickinson, Franklin Lakes, NJ, USA). The remaining suspension was inoculated into blood agar, MacConkey agar, chocolate agar, and Schindler’s agar for anaerobic culture.

Fungal cultures were obtained using Sabouraud dextrose agar. Additionally, a germ tube test was used to identify *Candida albicans*, and an API 20C AUX strip (bioMérieux, Paris, France) was used to identify other *Candida* species. Mueller-Hinton agar with 2% glucose was used for antifungal susceptibility testing. A breakpoint reference recommended by the Clinical and Laboratory Standards Institute (CLSI M27-S4) was used to determine the minimal inhibitory concentration for antifungal susceptibility of yeasts.

All patients underwent surgical catheter insertion and received antibiotic prophylaxis at catheter implantation. As per ISPD guidelines, topical antibiotics were applied to the catheter exit site for all patients. Peritonitis-related death was recorded if death occurred during the peritonitis treatment course.

All FP episodes were matched at a 1:4 ratio with PD-related bacterial peritonitis episodes. All control patients were diagnosed and hospitalized in the same institution and were under 14 years of age.

A data collection sheet was designed for this study, consisting of 3 sections that included 33 items. Count with percentage was determined for patient demographics, primary diagnosis, comorbidities, laboratory parameters, and outcomes. Fisher’s exact test was used for comparisons of percentages between the FP and bacterial peritonitis groups. We used SPSS software (version 26, IBM, Armonk, NY, USA) for statistical analyses. A *p* value < 0.05 was considered statistically significant.

## Results

A total of 194 episodes of PD-related peritonitis occurred between 2007 and 2017, among which 11 were FP episodes, representing a rate of 0.03 episodes per patient-year. Of the 10 patients, 7 of the patients were females.The patients age at FP episodes were 0–12 months (2 episodes) 13–36 months (6 episodes), and more than 36 months (3 episodes). The mean age at FP was 38 months (standard deviation, 35.9). Also, 9 patients had a single episode, and 1 (patient 9) had 2 episodes (Table 1). The primary diagnoses of the 10 patients were as follows: congenital nephrotic syndrome in 4 patients, infantile nephrotic syndrome in 2 patients, polycystic kidney disease in 2 patients, hypoplastic kidney in 1 patient, and a single dysplastic kidney in 1 patient. The most frequent comorbidities were hypertension (6 patients), congenital cardiac disease (6 patients), and liver and neurological diseases (3 patients).

**Table 1:** Patients primary diagnosis and microbiological characteristics of each FP episode (*n* = 11)

Primary Diagnosis	Time between PD insertion and first FP (Months)	Previous Peritonitis	Anti-fungal Prop.	G Tube	Culture and Sensitivity	Duration of Therapy (Weeks)	Antimicrobial Treatment	Route of Antibiotic Administration	Outcome of Peritonitis	Antibiotic Use 1 Month Prior to FP Episode
SDK	1	Yes	Yes	No	*Candida albicans*	3	Caspofungin	IV	CRS-HD	Yes
CNS	28	Yes	No	No	*Rhodotorula*	2	Amphotericin B	IV	CRS-HD	No
CNS	37	Yes	No	No	*Candida parapsilosis*	2	Fluconazole	IV/IP	CRS-HD-D	No
INS	20	Yes	No	Yes	*Aspergillus flavus*	3	Voriconazole	PO	CRS-HD^a^	No
PKD	36	Yes	Yes	No	*Candida famata*	3	Amphotericin B	IV	CRS-HD^a^	Yes
HPK	20	No	No	No	*Candida lusitaniae*	3	Caspofungin	IV	CRS-HD	No
INS	2	Yes	Yes	Yes	*Candida albicans*	2	Fluconazole	IV	CRS-HD	Yes
CNS	14	No	No	No	*Candida dubliniensis*	2	Fluconazole	IV	CRS-HD	No
CNS^b^	12	No	No	No	*Candida parapsilosis*	2	Fluconazole	IV/IP	CRS-HD^a^	No
CNS^b^	16	Yes	No	No	*Candida tropicalis*	3	Fluconazole	IV/IP	CRS-HD	No
PKD	21	Yes	Yes	No	*Candida famata*	4	Caspofungin	IV	CRS-HD	Yes

^a^ Temporary haemodialysis

^b^ same patient had 2 FP episodes

*FP* fungal peritonitis, *G tube* gastrostomy tube, *ep* episode, *SDK* single dysplastic kidney, *CNS* congenital nephrotic syndrome, *INS* infantile nephrotic syndrome, *HPK* hypoplastic kidney, *PKD* polycystic kidney disease, *prop.* prophylaxis, *CRS-HD* catheter removed and patient shifted to haemodialysis, *D* death, *IV* intravenous, *IP* intraperitoneal, *PO* oral

The clinical presentation consisted of fever in 7 episodes, abdominal pain in 6 episodes, cloudy effluent in 5 episodes, diarrhoea in 4 episodes, and vomiting in 4 episodes. No associated bloodstream infections were documented during the 11 episodes.

Eight of the episodes were preceded by an incidence of peritonitis, while the remaining 3 occurred as the first episode of peritonitis. In all patients, the white blood cell count in the PD effluent was greater than 100/μL and the neutrophil count was greater than 50%. Prior antibiotic use within 1 month of an FP episode was recorded in 4 episodes. All these patients had received antifungal prophylaxis, and 2 of them (patients 4 and 7) had a gastrostomy tube. The identified microorganisms included *Candida albicans* (2 episodes), *C. famata* (2 episodes), *C. parapsilosis* (2 episodes), *C. dubliniensis* (1 episode), *C. lusitaniae* (1 episode), *C. tropicalis* (1 episode), *Rhodotorula* species (1 episode), and *Aspergillus flavus* (1 episode). Antifungal susceptibility results are shown in Table 2*.*

**Table 2:** Antifungal Susceptibility (*n* = 11)

C/S	Fluconazole	Itraconazole	Voriconazole	Amphotericin	Caspofungin
*Candida albicans*	R	ND	S	S	S
*Rhodotorula*	ND	ND	ND	ND	ND
*Candida parapsilosis*	S	S	S	S	S
*Aspergillus flavus*	ND	ND	ND	ND	ND
*Candida famata*	S	S	ND	S	ND
*Candida lusitaniae*	I	S	1	S^a^	S
*Candida albicans*	S	S	S	S	S
*Candida dubliniensis*	S	S	S	S	S
*Candida parapsilosis*	S	R	S	S	S
*Candida tropicalis*	S	S	S	S	S
*Candida famata*	I	R	ND	S	S

^a^With intrinsic resistance

*ND* no data, *R* resistant, *S* sensitive, *I* intermediate

Most patients received 2–3 weeks of therapy, except for 1 patient who underwent 4 weeks of therapy. With respect to antifungal therapy, 5 patients received fluconazole, 3 received caspofungin, and 2 received amphotericin B. The patient with *A. flavus* infection received voriconazole. The routes of antifungal administration were intravenous in 6 episodes, intravenous and intraperitoneal in 4 episodes, and oral in 1 episode. In all episodes, the catheters were removed, and the patients were shifted to haemodialysis. For 3 patients, haemodialysis was temporary and was followed by a resumption of PD. Unfortunately, 1 patient died during peritonitis treatment despite catheter removal (Table 1).

A comparison of the demographic characteristics, primary diagnosis, comorbidities, laboratory results, and clinical outcomes between patients with FP and bacterial peritonitis is shown in Table 3. We found a higher proportion of patients younger than 5 years of age in the FP group. Additionally, the FP group had a higher proportion of patients with congenital/infantile nephrotic syndrome and patients with liver diseases as a comorbidity (*p* = 0.005). PD duration was associated with fungal peritonitis in the first 12 months of dialysis. Regarding the peritonitis outcome, patients with FP had a higher rate of catheter removal than those with bacterial peritonitis (*p* <  0.001). There was no significant difference in sex, effluent analysis results, or serum laboratory test findings between the groups.

**Table 3:** comparison between fungal and bacterial peritonitis

Variable	FP *n* = 11 (%)	BP *n* = 44 (%)	*P value*
*Gender*			
M	3 (27.3)	25 (56.8)	0.100
F	8 (72.7)	19 (43.2)	0.100
*Age at peritonitis episode (years)*			
Less than 5	10 (90.9)	14 (31.8)	**0.001**
More than 5	1 (9.1)	30 (68.2)	**0.001**
*Comorbidities*			
Hypertension	8 (72.7)	30 (68.2)	1.000
Cardiac diseases	7 (63.6)	15 (34.1)	0.094
Neurological diseases	3 (27.3)	10 (22.7)	0.709
Liver diseases	3 (27.3)	2 (4.5)	**0.049**
*Primary diagnosis*			
CNS/INS	7 (63.6)	8 (18.2)	**0.005**
HPK	1 (9.1)	2 (4.5)	0.495
PKD	2(18.2)	9 (20.5)	1.000
*PD duration (months)*			
0_12	9 (81.8)	12 (27.3)	**0.001**
13_24	0 (0)	7 (15.9)	0.323
More 24	2 (18.2)	26 (59.1)	**0.02**
*Effluent analysis*			
WCC > 100/μL	11 (100)	37 (84.1)	0.323
PMN > 50%	11 (100)	39 (88.6)	0.571
*Blood tests*			
Sodium > 135 (mmol/L)	5 (45.5)	12 (27.3)	0.286
Potassium> 3.5 (mmol/L)	4 (36.4)	19 (43.2)	0.745
Albumen > 25 (g/L)	4 (36.4)	26 (59.1)	0.198
*Outcome*			
Resolved	0 (0)	35 (79.5)	**< 0.001**
Catheter removed	10 (90.9)	9 (20.5)	**< 0.001**
Death	1 (9.1)	0 (0)	0.200

*FP* fungal peritonitis, *BP* bacterial peritonitis, *M* male, *F* female, *SDK* single dysplastic kidney, *CNS* congenital nephrotic syndrome, *INS* infantile nephrotic syndrome, *HPK* hypoplastic kidney, *PKD* polycystic kidney disease, *PD* peritoneal dialysis, *WCC* white cell count, *PMN* Polymorphonuclear neutrophils

## Discussion

FP is an infrequent but serious complication of PD and is concomitant with a high percentage of temporary or permanent membrane failure requiring haemodialysis. Furthermore, increased rates of hospitalization, morbidity, and mortality are reported in cases of FP. The initial presentation of FP is similar to that of peritonitis caused by bacterial organisms, which hinders early diagnosis when fungal culture is not performed [13]. To our knowledge, this is the first study describing FP and antifungal susceptibility in paediatric patients undergoing PD in Saudi Arabia. Moreover, the literature on the prevalence and outcomes of FP in children is limited.

Recently, Munshi et al. described FP outcomes of patients enrolled in the Standardizing Care to Improve Outcomes in Paediatric End Stage Renal Disease (SCOPE) Collaborative. They found that almost 60% of patients were younger than 2 years of age and gastrostomy tube was used in 50% of FP patients [4]. In the current study, we found that children below 5 years of age formed the majority of FP patients and gastrostomy was used only in 2 patients. Bladder and bowel incontinency might represent a risk for infection in the younger population.

In the North American Paediatric Renal Trials and Collaborative Study (NAPRTCS), the largest study on FP in PD patients aged 21 years or younger, Warady et al. found recent antibiotic use was observed in 56% of episodes [8].. In our study, recent antibiotic use was observed in only 36% of all episodes. However, 3 FP episodes in our study were not proceeded by bacterial peritonitis that requires antibiotics, which might be the reason for low recent antibiotic use. Recent antibiotic exposure could predispose patients to FP, as demonstrated in some adult and paediatric observational studies. This susceptibility may be explained by the overgrowth of fungi in the gastrointestinal system due to antibiotic use, which leads to transmural migration of fungi to the peritoneal cavity [14, 15].

Published study showed different rate of FP among paediatric patients on PD. In our study, FP rate was 5.6%, lower than the rate of the Iranian and SCOPE studies (12.9 and 8%, respectively) [4, 8, 16, 17]*.* We believe that the FP rate may be different from region to region due to various risk factors and access to medical care.

Interestingly, in the current study, the primary diagnoses of congenital, and infantile nephrotic syndrome were the most common primary diagnosis, which is significantly different from the observation in patients with bacterial peritonitis. Patients with congenital nephrotic syndrome have higher risks of infection and PD-related peritonitis than other patients with renal disease. The early start of PD in patients with congenital nephrotic syndrome makes them susceptible to frequent infections. Other risk factors include albumin loss and low immunoglobulin concentrations. However, Dufek et al. reported that most cases of PD-related peritonitis in patients with congenital nephrotic syndrome are bacterial, and the peritonitis rate in these patients is 0.77 per patient-year. In an observational study, invasive candidiasis was found in 3% of patients with congenital nephrotic syndrome who had started PD before 1 year of age, and 5 out of 7 patients had previous bacterial peritonitis before an FP episode [18, 19]. However*,* low albumin concentration was not significantly different between fungal and bacterial peritonitis, which has been linked with a risk of technique failure [20].

In our study, peritonitis episodes caused by *Candida* species and the non-*albicans* types were the most predominant. Almooosa et al. found that the most common invasive *Candida* species in paediatric patients was *C. albicans,* which accounted for 53% of cases, while *C. parapsilosis* (21%) and *C. tropicalis* (10%) were the most predominant non-*albicans* species in the same centre*.* Susceptibility testing for invasive candidiasis revealed a high susceptibility of *Candida* species to amphotericin B (97.6%), followed by caspofungin (96.3%), and fluconazole (78%). A similar finding was observed in our peritoneal fluid *Candida* species isolates: susceptibility to amphotericin B and caspofungin was 100%, while susceptibility to fluconazole was 78%. Surprisingly, a Canadian study reported 9 episodes of FP due to *Candida* species, in which 3 isolates (*C. tropicalis*, 1; *C. parapsilosis*, 1; *C. glabrata*, 1) were resistant to amphotericin B [21, 22]. Understanding the regional fungal susceptibility is essential for early administration of appropriate antifungals, which has an impact on the outcome of peritonitis.

In this study, catheter removal was necessary in 91% of episodes, which is consistent with the findings of the SCOPE and NAPRTCS studies*.* In a previous retrospective study, the FP mortality rate was 2.9%, while we observed only 1 death during FP [4, 22]. The mortality rate in the adult population with FP was reported to be higher than that in the paediatric age group (15–50% vs. 0–6%, respectively) [16]*.*

Current guidelines recommend starting antifungal therapy and prompting PD catheter removal, which could improve the outcome of PD-related FP episodes because knowing the local epidemiology of a fungal infection is very helpful for the empirical use of antifungals. Antifungal prophylaxis during bacterial peritonitis treatment is still controversial, although it is recommended in ISPD guidelines based on randomized trials [3, 23, 24].

The retrospective design and the possibility of potential bias in the matching of fungal to bacterial peritonitis using the peritoneal dialysis database are some limitations of this study. Additionally, the small number of FP episodes did not allow the use of multivariate analysis. Nonetheless, this is the first study reporting PD-related FP occurrence in Saudi Arabia.

## Conclusions

This study revealed that FP is associated with a significant risk of peritoneal membrane failure among children undergoing PD. Therefore, early diagnosis and prompt management are essential. We also found that congenital/infantile nephrotic syndrome and young age (5 years old or younger) were risk factors for FP in children undergoing PD.

This was a single-centre study; hence, our findings cannot be generalized. Moreover, due to the rarity of FP, the total number of cases was limited. A multicentre, prospective cohort study is recommended for better identification of the risk factors and outcomes of PD-related FP.

## Data Availability

The data and material of the study are available from the corresponding author MA on request.

## Abbreviations

FP: Fungal Peritonitis
PD: Peritoneal Dialysis
ISPD: International Society for Peritoneal Dialysis
SCOPE: Standardizing Care to Improve Outcomes In Paediatric End Stage Renal Disease
NAPRTCS: North American Paediatric Renal Trials and Collaborative Study
CLSI: Clinical and Laboratory Standards Institute
SPSS: Statistical Package for The Social Sciences
